# Selected hallmarks of hallux valgus in older women with symptomatic hallux valgus compared to middle-aged women with and without deformation of the forefoot

**DOI:** 10.1038/s41598-022-23113-z

**Published:** 2022-10-31

**Authors:** Agnieszka Jankowicz-Szymańska, Katarzyna Wódka, Marta Bibro, Eliza Smoła, Aneta Bac

**Affiliations:** 1 Faculty of Health Sciences, University of Applied Sciences in Tarnow, ul. Mickiewicza 8, 33-100 Tarnów, Poland; 2 Faculty of Motor Rehabilitation, Bronislaw Czech University of Physical Education in Krakow, Krakow, Poland

**Keywords:** Health care, Risk factors

## Abstract

The aim of the study was to compare the shape of the feet, the mobility of the metatarsophalangeal and interphalangeal joints and the flexibility of the calf muscles in older women with hallux valgus versus middle-aged women with and without this deformation to identify the presence of features which correlate particularly strongly with hallux valgus, and on which prophylaxis and conservative treatment should focus. The study involved 201 women: 92 aged 60–84 years with hallux valgus of both toes, 78 aged 38–59 with hallux valgus of both toes, and 31 aged 38–57 years with correctly shaped feet. The intensity of pain in the foot, the valgus angle of the big toe and fifth toe, the longitudinal and transverse arches of the foot, the symmetry of foot load with body weight, toe joint mobility and muscle flexibility were analysed. Both groups of women with hallux valgus differed from women with normal feet in the height of the transverse arch, the extent of dorsal extension in the first metatarsophalangeal joint and plantar flexion in the first interphalangeal joint. Older women were additionally characterised by reduced plantar flexion in the metatarsophalangeal joint of the big toe, limited flexibility of the soleus and gastrocnemius muscles as well as less pain in the toe area than in the foot itself. The most characteristic changes which were observed in older women with hallux valgus are a limited range of motion in the MTP and IP joints of the big toe, a reduced transverse arch and increased restriction of calf muscle flexibility.

## Introduction

Hallux valgus is among the most common causes of orthopaedic advice and foot surgery^1^. This deformity is the reason for up to 50% of visits to a doctor during which patients report a problem with the feet^2^. Hallux valgus is characterised by the incorrect position of the big toe in relation to the first metatarsal bone. An abnormal rotation and angle appear between the proximal phalanx and the metatarsal bone (the big toe is abducted towards the other toe)^3^.

The prevalence of hallux valgus is estimated at 23% to 64% depending on the gender, age and country of origin of the studied population^4–6^. Hallux valgus is more than twice as common in women as in men, more often in older people, and, importantly, rarely affects only one foot^7^. Some studies show a correlation between hallux valgus and excess body weight^8,9^. In most cases, patients with hallux valgus admit that they have a familial deformity, which confirms the theory about the genetic background of the lesion^10^. Some studies have shown that certain specific anatomies of the foot predispose to the development of hallux valgus, such as a rounder shaped head of the first metatarsal bone^11^, while the relationship between arching of the foot and hallux valgus remains controversial^12,13^. The causes of hallux valgus of the big toe are also seen in environmental factors, the most frequently mentioned of which is the wearing of shoes with a narrow front and high heels^10^.

Hallux valgus is not only an aesthetic deformity, but can lead to a number of serious disorders such as pain^7,14,15^, an abnormal gait pattern and deterioration of balance, increasing the risk of falling and, finally, reducing the quality of life^16–18^. Among the daily difficulties caused by foot pathologies, patients often mention the difficulty of choosing comfortable shoes and the limiting leaving the house^19^. The degree of quality-of-life deterioration is correlated with the severity of hallux valgus deformity^20^. For the above-mentioned reasons, hallux valgus (although it is not listed among the most serious contemporary diseases or those burdened with the greatest socio-economic effects) should be the subject of research and discussions aimed at better understanding the aetiology and pathomechanism of this deformity and, more importantly, developing the principles of effective prevention and treatment, including conservative treatment^21,22^. The aim of our study was to compare the intensity of pain, the shape of the feet, the mobility in the metatarsophalangeal (MTP) and interphalangeal (IP) joints of the toe and the flexibility of the calf muscles in women over 60 years of age with hallux valgus and in younger women with and without this deformation. Research shows that asymmetry of lower limb loading reduces postural stability by worsening the working conditions of the hip joint and increasing the setting movements of the ankle joint. This can cause falls, which is why it was decided to include the lower limb weight loading symmetry test in the study^23^. The authors hoped that this analysis would help identify the presence of features which correlate particularly strongly with hallux valgus, and on which prophylaxis and conservative treatment should focus.

## Material and methods

The study involved 201 women aged 38 to 84 years. All women were diagnosed for the presence of hallux valgus by an orthopaedic doctor who used the Manchester scale. This diagnostic tool is a set of 4 standardized photographs showing the 4 grades of hallux valgus (no deformity, mild deformity, moderate and severe deformity) to which the patient's deformity is compared. The reliability, reproducibility and comparability of the hallux valgus assessment with x-ray diagnosis has been scientifically proven^24,25^. The results of the Manchester scale are shown in Table 1.

**Table 1:** Degree of hallux valgus deformity in individuals from each group.

Group	Manchester scale			
	A, n (%)	B, n (%)	C, n (%)	D, n (%)
**G1 (60 + HV)**				
Right foot	0 (0%)	56 (61%)	24 (26%)	12 (13%)
Left foot	0 (0%)	49 (53%)	28 (30%)	15 (16%)
**G2 (< 60 HV)**				
Right foot	0 (0%)	56 (72%)	22 (28%)	0 (0%)
Left foot	0 (0%)	53 (68%)	25 (32%)	0 (0%)
**G3 (< 60)**				
Right foot	31 (100%)	0 (0%)	0 (0%)	0 (0%)
Left foot	31 (100%)	0 (0%)	0 (0%)	0 (0%)

The women were divided into three groups: G1—92 women (45.77%) aged 60 and over, with diagnosed hallux valgus in both feet (68.42 ± 6.36 years); G2—78 women (38.81%) under the age of 60, with diagnosed hallux valgus in both feet (50.62 ± 6.24 years); G3—31 women (15.42%) under the age of 60 whose toes were in the correct position in both feet (47.03 ± 4.61 years). Only women who did not complain of foot pain were eligible for group G3, assuming that the presence of pain could indicate an undiagnosed pathology, such as Morton’s neuroma. All the examined women were volunteers who responded to an advertisement about the study. The conditions for exclusion from the study were: abnormal position of the big toe in only one-foot, neurological diseases, gout, rheumatoid arthritis, diabetic foot, previous foot surgery, and lower limb trauma in the two months prior to the test.

- The women were informed in detail about the purpose and course of the study and signed consent to participate in it. The study was approved by the Bioethics Committee at the Regional Medical Chamber in Tarnów (Poland) (No 4/0177/2016) and the Australian New Zealand Clinical Trials Registry (ACTRN12621000902897). All the requirements of the Declaration of Helsinki for research involving human participants were respected during the conduct of the study.
- In order to determine the body weight status of the examined women, height (scaled anthropometer) and body weight (TANITA BF 350 total body composition analyser) were measured. Using the standard formula, the body mass index (BMI) was calculated and, in accordance with WHO recommendations, overweight was diagnosed when the value of this indicator was greater than 25 kg/m^2^, and obesity when the BMI was greater than 30 kg/m^2^. Using the same TANITA scale, the percentage of body fat was determined. The women in the G1 and G2 groups were asked to rate the intensity of pain in the foot and hallux valgus (separately) on a scale of 0 to 10 (the numeric pain rating scale—NPR scale)^26^. The question was about the greatest pain the women had experienced in the past month.
- The plantar side of the feet was examined in both feet while standing barefoot using an electronic podoscope (CQ Elektronik System). The plantoconturograms obtained were used to analyse the foot length (measured from the longest toe to the back of the heel), foot width (measured at the widest point of the forefoot), alpha—hallux valgus angle (the angle between the tangent to the medial edge of the foot and the tangent to the medial edge of the big toe; the correct value of the alpha angle is from 0° to 9°)^27^, beta—the varus angle of the fifth toe (the angle between the tangent to the lateral edge of the foot and the tangent to the lateral edge of the fifth toe; the correct value of the beta angle is from 0° to 5°), gamma—the heel angle (the angle between the tangent to the medial edge of the foot and the tangent to the lateral edge of the foot, the correct gamma angle is from 15° to 18°^27^, values greater than 18° indicate transverse planar feet) and Clarke’s (the angle between the tangent to the medial edge of the foot and the line joining the point of the greatest indentation and the contact of the medial tangent with the edge of the forefoot [values lower than 42° indicate a longitudinal flat foot, and values greater than 54° a hollow foot])^28^ angles, and the Wejsflog index (the proportion of the length to the width of the foot, values closer to 3 indicate the correct transverse arch of the foot, values closer to 2 a transverse flatfoot).

Additionally, a baroresistive platform (P-walk, BTS Bioengineering) was used to examine the load of the body weight on the right and left lower limbs (the results were given in percentage points); the difference in the weight load on the feet and the arch index (AI) were estimated (the ratio of one-third of the area of the middle plantoconturogram to the area of the entire plantoconturogram without toes, the correct value of the index is 0.21–0.28, values above 0.28 indicate longitudinal flat feet)^29^. Both the podoscope and the platform are reliable diagnostic tools that are repeatedly used in scientific research, also on the group of elderly people^30–36^.

The ranges of flexion and extension of the MTP joint of the big toe and flexion in the IP joint were also examined using a goniometer. Extension of the MTP joint was examined in a sitting position with the feet flat on the ground. Flexion of the MTP and IP joints was examined in the supine position on the side of the tested limb^37^. When examining the range of motion in the MTP joint, the goniometer arms were positioned along axis I of the metatarsal bone and the axis of the proximal phalanx. While examining the IP joint, the arms of the goniometer were placed along the axis of the proximal phalanx and the distal phalanx.

**Figure 1:**
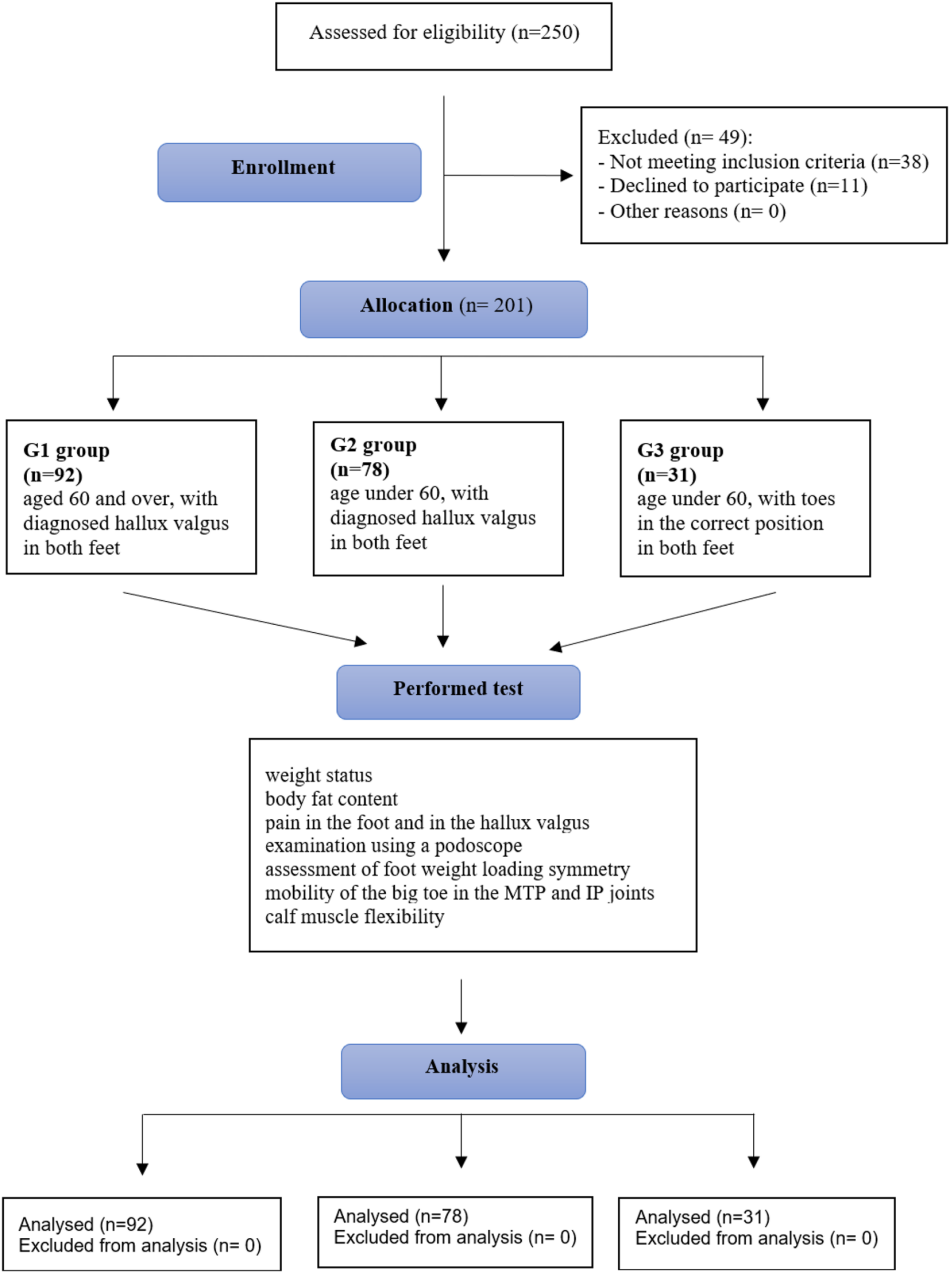
Scheme of research.

The elasticity of the gastrocnemius and soleus muscles was tested in the supine position, first with the lower limbs extended and the feet off the edge of the bed, then with the test limb bent at the hip and the knee joint at approximately 90°. The range of motion of both muscles was considered correct if the therapist was able to position the patient’s foot at right angles to the lower leg (negative test result) in both positions, without using excessive force, pulling the heel with one hand and lightly pressing on the plantar side towards the head with the other. If this was not possible, with both a bent and a straightened knee, the therapist diagnosed a shortening of the gastrocnemius and soleus muscles. If the knee extension test was positive and the knee flexion test was negative, only the gastrocnemius was shortened^38^. The tests were conducted by experienced physiotherapists, the authors of the study. One person carried out tests on a podoscope for all participants, a second person carried out tests on a platform (symmetry of lower limb loading), and a third person assessed the elasticity of the calf muscles. Until the end of the study, the researchers did not know the results of the other tests. The procedure for recruiting and conducting the research is shown in the diagram (Fig. 1).

Statistical analysis was performed using Statistica v13 software. Frequency tables and basic descriptive statics were used. The normality of the distribution of variables was tested with the Shapiro–Wilk test and the homogeneity of variance with the F-test. The significance of intergroup differences was determined using the one-way ANOVA and the Tukey post hoc test, and the chi-square test was applied for qualitative variables. The level of significance was set at α = 0.05. Additionally, for pairs of quotient variables that were statistically significantly different, a standardised effect size was calculated (low values meant that the differentiation was of little importance, even if it was statistically significant, a value of about 0.5 meant moderate, and values of 0.8 and above a significant effect size). To investigate the relationship between the valgus angle of the big toe, the width of the foot and the size of the transverse arch, the Pearson correlation was used (correlations below 0.4 were considered weak, between 0.4 and 0.7 moderate, above 0.7 fairly strong, and above 0.9 too strong).

### Institutional review board statement

The study was conducted according to the guidelines of the Declaration of Helsinki, and approved by the Regional Medical Chamber (No 4/0177/2016).

### Informed consent statement

Informed consent was obtained from all subjects involved in the study.

## Results

The BMI of women in the G1 group was 28.20 ± 3.82 kg/m^2^, in the G2 group 26.30 ± 4.25 kg/m^2^, and in the G3 group 23.96 ± 3.45 kg/m^2^. A statistically significant difference in the BMI values was noted between the G1 group and the G2 (p < 0.001) and G3 (p < 0.001) groups. The amount of adipose tissue in the body was highest in the G1 group (36.56 ± 5.62%), slightly lower in the G2 group (31.12 ± 7.22%) and lowest in the G3 group (28.22 ± 7.73%). Significant differences were noted between the G1 group and the G2 and G3 groups.

The women from the G1 group rated the pain intensity in hallux valgus as 2.30 ± 2.82 on a scale of 0–10, and in the foot (except the toe area) as 3.30 ± 3.25. Those in the G2 group reported stronger pain in the big toe area—3.46 ± 2.89 (here a significant difference was noted between the G1 and G2 groups, p < 0.001) and of a similar intensity in the foot—3.33 ± 4.08 (no significant differentiation).

The alpha angle, showing the severity of the hallux valgus, was significantly greater in the G1 and G2 groups than in the G3 group, but its size in the group of older women diagnosed with the deformity (G1) was the same as in the group of younger women with the deformity (G2) (Table 2). The women in the G1 group, unlike those in the G2 and G3 groups, put more weight on the left lower limb. A significant difference, both in the load on the left and right lower limbs, was noted between the G1 and G2 groups. The difference in the load on the right and left lower limbs was greatest in the G2 group, slightly smaller (by 1.23%) in the G1 group and significantly smaller (by 3.65%) in the G3 group. Significant although weak correlations were observed between the intensity of pain in the hallux valgus and the difference in lower limbs loading with body weight. For the left foot, the correlation coefficient value was R = 0.33 at p < 0.01, for the right foot R = 0.27 at p < 0.01.

**Table 2:** Position of the big toe, the symmetry of the lower limb load and indices showing the longitudinal arch of the foot.

Variable	Group	Mean	Median	Min	Max	SD	p	Se
Alpha angle left foot (^o^)	G1 (60 + HV)	22.10	23.10	-0.40	52.70	13.05	G1 & G2 p = 0.998	na
	G2 (< 60 HV)	22.01	23.35	1.10	38.50	8.04	G1 & G3 p < 0.001*	1.59
	G3 (< 60)	6.11	6.10	1.90	9.00	2.09	G2 & G3 p < 0.001*	1.59
Alpha angle right foot (^o^)	G1 (60 + HV)	22.28	21.50	-2.70	54.50	13.35	G1 & G2 p = 0.584	na
	G2 (< 60 HV)	20.74	21.35	6.20	37.50	7.25	G1 & G3 p < 0.001*	1.57
	G3 (< 60)	6.54	7.10	0.40	9.00	1.93	G2 & G3 p < 0.001*	1.42
Left foot load (%)	G1 (60 + HV)	50.08	49.00	36.70	65.10	5.24	G1 & G2 p = 0.004*	0.24
	G2 (< 60 HV)	47.59	47.80	36.20	60.50	5.21	G1 & G3 p = 0.314	na
	G3 (< 60)	48.55	48.50	35.20	58.20	3.78	G2 & G3 p = 0.638	na
Right foot load (%)	G1 (60 + HV)	49.92	51.00	34.90	63.30	5.24	G1 & G2 p = 0.004*	-0.24
	G2 (< 60 HV)	52.41	52.20	39.50	63.80	5.21	G1 & G3 p = 0.315	na
	G3 (< 60)	51.45	51.50	41.80	64.80	3.78	G2 & G3 p = 0.639	na
Lower limb load difference (%)	G1 (60 + HV)	8.08	6.80	0.00	30.20	6.62	G1 & G2 p = 0.444	na
	G2 (< 60 HV)	9.31	8.00	0.20	27.60	6.66	G1 & G3 p = 0.176	na
	G3 (< 60)	5.66	4.80	0.20	29.60	5.72	G2 & G3 p = 0.023*	0.36
Arch Index left foot (%)	G1 (60 + HV)	24.96	25.59	2.39	36.64	6.10	G1 & G2 p = 0.394	na
	G2 (< 60 HV)	23.68	25.84	1.81	34.13	6.93	G1 & G3 p = 0.884	na
	G3 (< 60)	24.33	25.12	4.41	33.57	5.20	G2 & G3 p = 0.876	na
Arch Index right foot (%)	G1 (60 + HV)	24.90	25.26	5.11	34.65	5.40	G1 & G2 p = 0.499	na
	G2 (< 60 HV)	23.79	25.88	2.72	34.36	7.87	G1 & G3 p = 0.931	na
	G3 (< 60)	24.42	24.82	12.22	31.80	4.43	G2 & G3 p = 0.885	na
Clarke’s angle left foot (^o^)	G1 (60 + HV)	45.19	45.55	19.30	60.00	7.15	G1 & G2 p = 0.730	na
	G2 (< 60 HV)	44.42	44.25	25.80	59.30	6.37	G1 & G3 p = 0.661	na
	G3 (< 60)	43.99	44.10	31.50	58.10	5.89	G2 & G3 p = 0.951	na
Clarke’s angle right foot (^o^)	G1 (60 + HV)	45.37	45.95	25.70	62.50	7.59	G1 & G2 p = 0.712	na
	G2 (< 60 HV)	46.19	46.90	30.40	62.90	6.37	G1 & G3 p = 0.378	na
	G3 (< 60)	43.51	43.20	36.30	53.60	4.56	G2 & G3 p = 0.147	na

60 + HV—aged 60 years or older, hallux valgus; < 60 HV—aged less than 60 years, hallux valgus; < 60—aged less than 60 years

*Se *standardised effect, *na *not applicable.

*Statistically significant differences.

The effect size suggests that the observed intergroup differences were of little importance. None of the indices describing the longitudinal arch of the feet (neither the AI nor Clarke’s angle) selected for analysis showed any significant intergroup differentiation.

The examined women did not differ significantly in foot length (Table 3). The widths of the right and left feet were significantly greater (confirmed by the Es value) in women with hallux valgus compared to women with the correct toe position. Both indices showing the height of the transverse arch (the Wejsflog index and the gamma angle) were significantly different in the G1 and G2 groups compared to the G3 group and indicated a reduction in the transverse arch in women with hallux valgus, however, the value of Es indicates that this differentiation is of low significance. The beta angle showing the position of the fifth toe did not differ between the groups.

**Table 3:** Basic foot dimensions, transverse arch of the foot and the position of the fifth toe.

Variable	Group	Mean	Median	Min	Max	SD	p	Se
Left foot length (mm)	G1 (60 + HV)	230.27	231.45	203.00	260.00	11.27	G1 & G2 p = 0.971	na
	G2 (< 60 HV)	229.87	229.20	195.10	252.20	11.45	G1 & G3 p = 0.591	na
	G3 (< 60)	232.51	232.70	215.20	248.60	9.42	G2 & G3 p = 0.500	na
Right foot length (mm)	G1 (60 + HV)	230.23	231.45	200.60	261.00	11.59	G1 & G2 p = 0.961	na
	G2 (< 60 HV)	230.69	230.40	196.10	253.20	11.78	G1 & G3 p = 0.569	na
	G3 (< 60)	232.61	232.50	215.80	248.10	9.39	G2 & G3 p = 0.705	na
Left foot width (mm)	G1 (60 + HV)	101.31	101.15	83.00	118.90	8.22	G1 & G2 p = 0.947	na
	G2 (< 60 HV)	100.99	100.75	91.10	114.60	5.01	G1 & G3 p < 0.001*	0.82
	G3 (< 60)	93.10	93.50	83.50	101.40	4.57	G2 & G3 p < 0.001*	0.78
Right foot width (mm)	G1 (60 + HV)	102.43	102.25	88.10	119.10	6.48	G1 & G2 p = 0.391	na
	G2 (< 60 HV)	101.29	101.40	89.20	113.90	5.06	G1 & G3 p < 0.001*	0.73
	G3 (< 60)	95.13	95.60	86.30	103.10	4.29	G2 & G3 p < 0.001*	0.61
Wejsflog index left foot	G1 (60 + HV)	2.29	2.29	1.89	2.80	0.19	G1 & G2 p = 0.975	na
	G2 (< 60 HV)	2.28	2.26	1.99	2.63	0.15	G1 & G3 p < 0.001*	-0.02
	G3 (< 60)	2.50	2.49	2.25	2.83	0.12	G2 & G3 p < 0.001*	-0.02
Wejsflog index right foot	G1 (60 + HV)	2.25	2.26	1.93	2.53	0.16	G1 & G2 p = 0.325	na
	G2 (< 60 HV)	2.28	2.27	1.98	2.56	0.14	G1 & G3 p < 0.001*	-0.02
	G3 (< 60)	2.45	2.45	2.25	2.69	0.11	G2 & G3 p < 0.001*	-0.01
Beta angle left foot (^o^)	G1 (60 + HV)	15.74	16.10	0.40	41.90	6.58	G1 & G2 p = 0.962	na
	G2 (< 60 HV)	15.97	15.60	1.20	26.30	4.82	G1 & G3 p = 0.911	na
	G3 (< 60)	16.22	16.20	9.10	24.20	4.18	G2 & G3 p = 0.975	na
Beta angle right foot (^o^)	G1 (60 + HV)	17.17	16.40	1.40	103.00	10.96	G1 & G2 p = 0.400	na
	G2 (< 60 HV)	15.51	15.75	5.10	28.20	5.53	G1 & G3 p = 0.582	na
	G3 (< 60)	15.45	15.70	5.40	22.20	4.06	G2 & G3 p = 0.999	na
Gamma angle left foot (^o^)	G1 (60 + HV)	20.06	20.25	11.30	28.30	3.92	G1 & G2 p = 0.529	na
	G2 (< 60 HV)	20.62	20.75	13.00	27.50	3.14	G1 & G3 p < 0.001*	0.27
	G3 (< 60)	17.30	17.50	12.10	21.40	2.13	G2 & G3 p < 0.001*	0.33
Gamma angle right foot (^o^)	G1 (60 + HV)	20.51	20.20	14.10	29.30	3.74	G1 & G2 p = 0.768	na
	G2 (< 60 HV)	20.16	20.10	10.50	28.00	3.16	G1 & G3 p < 0.001*	0.32
	G3 (< 60)	17.29	17.50	13.20	21.20	1.98	G2 & G3 p < 0.001*	0.28

60 + HV—aged 60 years or older, hallux valgus; < 60 HV—aged less than 60 years, hallux valgus; < 60—aged less than 60 years.

*Se* standardised effect, *na* not applicable.

*Statistically significant differences.

In the G1 group, the range of flexion in the MTP joint of the big toe was almost two times smaller than in the G2 and G3 groups, in which the range of flexion was similar (Table 4). The range of extension in the MTP joint was the smallest in the G1 group, greater in the G2 group and the largest in the G3 group. The intergroup differences (except for the difference between G1 and G2 for the right foot) were statistically significant. The range of flexion in the IP joint of the big toe in both feet was significantly lower in the G1 group compared to the G2 and G3 groups and slightly smaller in the G2 group compared to the G3 group. The Es value confirms the clinical significance of the observed differences.

**Table 4:** Mobility of the big toe in the MTP and IP joints.

Variable	Group	Mean	Median	Min	Max	SD	P	Se
MTP plantar flexion left foot (^o^)	G1 (60 + HV)	23.77	25.00	0.00	57.00	9.69	G1 & G2 p < 0.001*	−1.83
	G2 (< 60 HV)	42.15	42.00	20.00	100.00	12.65	G1 & G3 p < 0.001*	−1.94
	G3 (< 60)	43.23	42.00	21.00	63.00	8.71	G2 & G3 p = 0.886	na
MTP plantar flexion right foot (^o^)	G1 (60 + HV)	21.04	20.00	5.00	50.00	8.81	G1 & G2 p < 0.001*	−1.92
	G2 (< 60 HV)	40.31	40.50	15.00	70.00	11.37	G1 & G3 p < 0.001*	−2.30
	G3 (< 60)	44.10	42.00	27.00	60.00	7.95	G2 & G3 p = 0.161	na
MTP dorsal extension left foot (^o^)	G1 (60 + HV)	38.21	38.80	3.00	74.50	14.82	G1 & G2 p = 0.147	na
	G2 (< 60 HV)	41.96	42.30	10.70	71.00	12.27	G1 & G3 p < 0.001*	−1.18
	G3 (< 60)	50.04	49.00	33.30	63.50	8.29	G2 & G3 p = 0.009*	−0.80
MTP dorsal extension right foot (^o^)	G1 (60 + HV)	36.61	36.35	3.80	72.20	13.51	G1 & G2 p < 0.001*	−0.78
	G2 (< 60 HV)	44.45	44.15	12.60	67.30	10.53	G1 & G3 p < 0.001*	−1.37
	G3 (< 60)	50.38	50.10	31.00	71.00	8.86	G2 & G3 p = 0.046*	−0.59
IP plantar flexion left foot (^o^)	G1 (60 + HV)	26.02	26.50	0.00	60.00	13.09	G1 & G2 p < 0.001*	−1.29
	G2 (< 60 HV)	37.04	38.00	0.00	77.00	15.05	G1 & G3 p < 0.001*	−1.75
	G3 (< 60)	43.61	42.00	9.00	72.00	13.31	G2 & G3 p = 0.067	na
IP plantar flexion right foot (^o^)	G1 (60 + HV)	26.26	30.00	0.00	55.00	12.53	G1 & G2 p < 0.001*	−1.26
	G2 (< 60 HV)	38.92	40.00	2.00	75.00	15.53	G1 & G3 p < 0.001*	−1.82
	G3 (< 60)	44.52	48.00	8.00	70.00	13.87	G2 & G3 p = 0.142	na

60 + HV—aged 60 years or older, hallux valgus; < 60 HV—aged less than 60 years, hallux valgus; < 60—aged less than 60 years.

*MTP *metatarsophalangeal joint, *IP* interphalangeal joint, *Se* standardised effect, *na* not applicable.

*Statistically significant differences;.

Correlations ranging from moderate to quite significant were found between the value of the alpha angle and the width of the forefoot as well as the values of the indices reflecting the height of the transverse arch of the foot: the Wejsflog index and the gamma angle (Table 5).

**Table 5:** Correlations between the angle showing the magnitude of hallux valgus deformity and the foot width and transverse arching indices.

Correlated variables	Alpha angle left foot (o)		Alpha angle right foot (o)	
	R	p	R	p
Left foot width (mm)	0.51	0.001*	0.49	0.001*
Right foot width (mm)	0.42	0.001*	0.64	0.001*
Wejsflog index left foot	−0.63	0.001*	−0.56	0.001*
Wejsflog index right foot	−0.52	0.001*	−0.72	0.001*
Gamma angle left foot (o)	0.61	0.001*	0.45	0.001*
Gamma angle right foot (o)	0.46	0.001*	0.64	0.001*

*Statistically significant correlations.

The clinical study showed a significantly more frequent occurrence of reduced elasticity of the soleus muscle in the G1 group compared to the G2 and G3 groups, and of the gastrocnemius muscle in the G1 group compared to the G2 group (Table 6).

**Table 6:** Elasticity of the calf muscles in women from the studied groups.

Muscle	Group	Left leg, n (%)		p
		Normal	Shortened	
Soleus	G1 (60 + HV)	24 (26.09%)	68 (73.91%)	G1 & G2 p < 0.001*
	G2 (< 60 HV)	41 (52.56%)	36 (46.15%)	G1 & G3 p = 0.003*
	G3 (< 60)	17 (54.84%)	14 (45.16%)	G2 & G3 p = 0.925
Gastrocnemius	G1 (60 + HV)	26 (28.26%)	64 (69.57%)	G1 & G2 p = 0.024*
	G2 (< 60 HV)	39 (50.00%)	39 (50.00%)	G1 & G3 p = 0.630
	G3 (< 60)	12 (38.71%)	19 (61.29%)	G2 & G3 p = 0.317

		Right leg, n (%)		
		Normal	Shortened	
Soleus	G1 (60 + HV)	24 (26.09%)	68 (73.91%)	G1 & G2 p < 0.001*
	G2 (< 60 HV)	43 (55.13%)	35 (44.87%)	G1 & G3 p = 0.003*
	G3 (< 60)	17 (54.84%)	14 (45.16%)	G2 & G3 p = 0.978
Gastrocnemius	G1 (60 + HV)	25 (27.17%)	67 (72.83%)	G1 & G2 p = 0.006*
	G2 (< 60 HV)	37 (47.44%)	41 (52.56%)	G1 & G3 p = 0.225
	G3 (< 60)	12 (38.71%)	19 (61.29%)	G2 & G3 p = 0.408

*Statistically significant differences.

## Discussion

Our observations show that the most characteristic changes accompanying hallux valgus in older women are a limited range of motion in the MTP and IP joints of the big toe and a reduced transverse arch.

Kim et al.^39^ also observed a reduced range of motion of the big toe in people with hallux valgus. They examined the range of motion of the big toe and hindfoot when walking and found that it decreased with increasing hallux valgus. The observations of Kawakami et al.^40^ who noted hypermobility of the forefoot in the late stage of walking in people with hallux valgus, are the opposite. According to Suh et al.,^41^ restricted motion in the first metatarsal joint may be the result of flatfoot and lead to the elevation of the first metatarsal head. This in turn limits the ability of the peroneus longus muscle to stabilise the first ray and contributes to its hypermobility. Such hypermobility was observed, for example, by Dietze et al.^42^ and Singh et al.^43^. It is often cited as a contributing factor in hallux valgus.

The results of research on the connection between hallux valgus and flatfoot are inconclusive. On the one hand, there are reports confirming the relationship between flatfoot and hallux valgus^40,44–46^; on the other hand, there are studies (for example, the already mentioned Suh et al.^41^) that contradict this correlation. In our study, no relationship was found between the longitudinal arch of the foot and hallux valgus of the big toe. However, we showed a significant correlation between the lowering of the transverse arch and hallux valgus. The transverse arch plays an important role in the upright position. It helps to adjust the foot’s shape to the ground surface and acts as a spring to absorb some of the forces while walking and running^47^. A lowered transverse arch may cause excessive foot pronation, which in turn may contribute to hindfoot valgus and hallux valgus^48,49^. Nakai et al.^50^ studied the transverse arch of the feet of people with and without hallux valgus. They found that in people with the deformity, the heads of the metatarsal bones were lowered and the sesamoid bone was rotated and tilted outwards. In our study, the lowering of the transverse arch in women with hallux valgus was evidenced by both significantly greater width of the feet, as well as by the values of the Wejsflog index and the gamma angle. The lack of a transverse arch may cause pain in the foot area, which was even stronger in the older women examined than the pain of the deformed toe itself. Nakai et al.^50^ also indicate a relationship between a lowered transverse arch and pain in the forefoot in people with hallux valgus. It should be considered whether rebuilding the transverse arch could be an effective prophylaxis of hallux valgus. Attempts to treat hallux valgus of the I and II degrees were undertaken by Dygut et al.^51^ In their study, 41 people with hallux valgus used a transverse arch orthosis for 5–7 h a day for 1 year and achieved toe position correction. On the other hand, it should be remembered that the transverse arch is the least understood characteristic of the foot and its relationship with forefoot deformities remains controversial and requires further research^52^. For example, Weijers et al.^53^ who studied plantar pressure of the foot, believe that the forefoot bones form a geometric but not a functional arc. Keeping these controversies in mind, one should be cautious in interpreting existing results and continue research.

For example, in contradiction to the studies described above are the observations of Zeidan et al.^53^ who noted a higher transverse arch in women with hallux valgus compared to women with normal feet. They studied the height of the transverse arch in three positions: sitting, standing with symmetrical foot loading and standing with the body weight shifted onto the test foot. In each of these three positions, the transverse arch was higher in participants with a hallux valgus toe, with no significant difference between women with a painful and non-painful deformity.

Research into the anatomy of the foot shows that there is a biomechanical relationship between calf muscle tone and toe position. This is possible because the calcaneus is the site of attachment of both the Achilles tendon and the plantar aponeurosis. The link between shortened calf muscles and juvenile hallux valgus was pointed out by Barouk^54^. Similar are the observations of O'Reilly et al.^52^ who examined adults. In our study, elderly women with hallux valgus had significantly more frequent shortening of the soleus muscle compared to younger women with and without hallux valgus, significantly more frequent shortening of the gastrocnemius muscle compared to women with normal feet and slightly more frequent shortening of the gastrocnemius muscle compared to younger women with hallux valgus. A particularly marked difference was found in the soleus muscle, shortening of this muscle was present in 2/3 of elderly women with hallux valgus and less than half of younger women with or without deformity. The results of our observations, should be confirmed in subsequent studies. In the light of the analysis of the literature collected and presented above, it also seems justified to study the position of the hindfoot and the length and function of the peroneus longus muscle and the tibialis anterior muscle, which are directly responsible for plantar arching. In conclusion, research should be continued on the importance of lowering the transverse foot arching for the development of hallux valgus and the relationship of both disorders with calf muscle shortening. An important observation resulting from our research is the correlation between pain in the hallux valgus and asymmetric lower limbs loading, which, especially in the elderly, may increase the risk of falls and reduce the quality of life. Limitation of our research was the lack of a control group of elderly women without foot deformity. This limitation should be supplemented in the future.

## Conclusions

In our study population foot pain and pain in the toe area were reported in both groups of women with hallux valgus (younger than 60 years and 60 years or older). However, women aged 60 years and older were more likely to complain of foot pain, while younger women reported similar levels of pain in the foot and big toe area. There was a greater tendency for lower limb loading asymmetry in women with hallux valgus than in the control group, especially in women under 60 years of age. Additionally, significant correlations were observed between the intensity of pain in the hallux valgus and the difference in lower limbs loading. The transverse arch of the feet was significantly lower in both groups of women with hallux valgus compared with women without this deformity. The size of the longitudinal arch did not correlate with the valgus angle of the big toe. Women under 60 years of age with hallux valgus had a significantly reduced range of dorsal extension at the MTP joint and sole flexion at the IP joint of the big toe compared with women without the foot deformity. This restriction was greater in older women, who also had significantly reduced sole flexion at the MTP joint. Calf muscle shortening was more characteristic of women aged 60 years and older with hallux valgus. In younger women with or without hallux valgus deformity, the prevalence of calf muscle shortening was similar.

## Data Availability

The datasets used and/or analysed during the current study available from the corresponding author on reasonable request.
